# Therapeutic Targets in Chronic Lymphocytic Leukemia

**DOI:** 10.3390/cancers12113259

**Published:** 2020-11-04

**Authors:** Luca Laurenti, Dimitar G. Efremov

**Affiliations:** 1 Department of Hematology, Fondazione Policlinico Universitario A Gemelli IRCCS, 00168 Rome, Italy; 2 Molecular Hematology, International Centre for Genetic Engineering and Biotechnology, 34149 Trieste, Italy

## Introduction

Chronic lymphocytic leukemia (CLL) is a common B cell malignancy and is the most common type of adult leukemia in western countries. It is characterized by the progressive accumulation of autoreactive B lymphocytes that are driven to expand by the combined effects of extrinsic signals generated by the B cell receptor (BCR) and other microenvironmental stimuli and cooperating intrinsic genetic lesions [1]. Studies conducted over the past two decades have provided important advances in the understanding of the pathogenesis of the disease and have led to the development of novel targeted therapies that have improved patient survival [2,3].

The genetic lesions that underlie CLL are heterogeneous and include gross cytogenetic abnormalities and point mutations [4,5,6]. The most common genetic defect in CLL is the 13q14 abnormality, which can be detected in 55–65% of cases and typically involves the microRNAs *mir-15/16* and the long noncoding RNA *Dleu2*. Other relatively common genetic lesions include trisomy 12, deletions and/or inactivating mutations in *ATM* and *BIRC3* (both located on 11q22), deletions and/or inactivating mutations in *TP53* (located on 17p13), and mutations in *NOTCH1* and *SF3B1*, each of which are typically detected in 5–20% of the cases at diagnosis. In addition, recurrent genetic lesions in more than 30 other genes have been detected with frequencies ranging from 2–5% of the cases [7]. Most of the affected genes cluster into one of several distinct biological pathways, including cell cycle, DNA damage response, RNA metabolism, and BCR, Toll-like receptor (TLR), Notch and NF-kB signaling. However, the exact mechanisms through which these genetic lesions contribute to CLL development and progression and their relative clinical relevance are still largely unknown and require further study.

The extracellular signals that drive CLL include interactions with various cellular elements present in the lymph node tumor microenvironment, such as T cells, monocyte-derived nurse-like cells and stromal cells [8]. These cells provide proliferation and survival signals to the leukemic B cells by secreting different chemokines and cytokines or by expressing distinct surface receptors or adhesion molecules. In addition, the lymph node is the site where CLL cells are believed to primarily receive growth signals induced by stimulation of the leukemic BCRs with external (auto)antigen or stimulation of the leukemic TLRs with TLR ligands, such as unmethylated DNA [9,10].

The identification of the BCR as a major driving force in CLL prompted the development of multiple drugs that block signaling through this receptor. Most of these drugs target BTK or PI3K, although inhibitors of SYK, SRC family kinases and mammalian target of rapamycin (mTOR) have also demonstrated activity in clinical trials [11]. However, despite the high overall response rates, BCR inhibitors in the vast majority of patients induce only partial responses. In addition, acquired resistance to the BTK inhibitor ibrutinib occurs after prolonged treatment in a substantial proportion of patients, and this is more frequent in patients with TP53 mutations [12,13]. Acquired resistance to ibrutinib has been associated with mutations in BTK or its downstream target PLCG2, which disable ibrutinib’s capacity to irreversibly bind and inhibit BTK or allow for BTK-independent activation of downstream BCR signaling pathways, respectively [14]. However, these defects are frequently found in only a small fraction of the malignant clones at the time of progression, suggesting that other mechanisms are also involved [15]. Possible mechanisms could include lower dependence on BCR signals of leukemic cells with particular combinations of genetic lesions and/or adoptive changes in the kinome resulting in greater activation of compensatory pathways that substitute for the BCR signal. Such mechanisms have been shown to account for ibrutinib resistance in other B cell malignancies, but further research is needed to determine whether this is also the case of CLL [16,17,18,19,20].

The BCL-2 antagonist venetoclax is another drug that has demonstrated impressive therapeutic efficacy in patients with CLL, including patients with adverse prognostic parameters such as a *TP53* aberration or refractoriness to fludarabine [21,22]. However, similar to ibrutinib and other BCR inhibitors, this drug induces relatively few complete responses on its own. The inability of venetoclax to eradicate all CLL cells is believed to be due to survival signals that the leukemic cells receive in the lymph nodes from various microenvironmental stimuli. Among them, survival signals derived from the BCR, T cells and TLRs have been shown to confer venetoclax resistance by modulating the expression of the antiapoptotic proteins MCL-1, BCL-xL and/or BFL-1 [23,24,25,26]. Drugs that can interfere with these signals, such as BTK, PI3K or SYK inhibitors, have been shown to sensitize the malignant cells and synergize with ventoclax in preclinical models. Moreover, recent clinical studies have shown substantially higher complete response rates with the combination of venetoclax and ibrutinib compared to each agent alone [27,28,29]. However, despite the improved efficacy of the combination, many patients still remain with residual disease or develop resistance, suggesting that additional drugs that target microenvironmental signals need to be evaluated to identify the optimal partner for venetoclax combination therapy.

Mutations in BCL-2 that reduce venetoclax binding affinity have emerged as another important mechanism of resistance to this drug [30,31,32]. However, other genetic lesions, such as mutations in *BTG1*, deletions of *CDKN2A/B* and amplification of *MCL-1*, have also been implicated in venetoclax resistance [33,34]. In addition, *TP53*, *NOTCH1*, and *SF3B1* mutations have been associated with shorter duration of response and/or reduced likelihood of achieving a CR, suggesting that multiple genetic lesions may influence sensitivity to venetoclax treatment [35]. Additional studies are needed to fully understand whether and how these genetic lesions affect the response to venetoclax and whether they can represent novel therapeutic targets for rational combination strategies to enhance venetoclax activity.

This Special Issue of *Cancers* focuses on the cell-intrinsic and cell-extrinsic mechanisms that mediate treatment resistance in CLL and on novel therapeutic approaches to overcome this resistance. A greater understanding of these mechanisms and identification of novel therapeutic targets may ultimately result in the development of personalized combination treatments that will further improve the outcome of patients with CLL.
